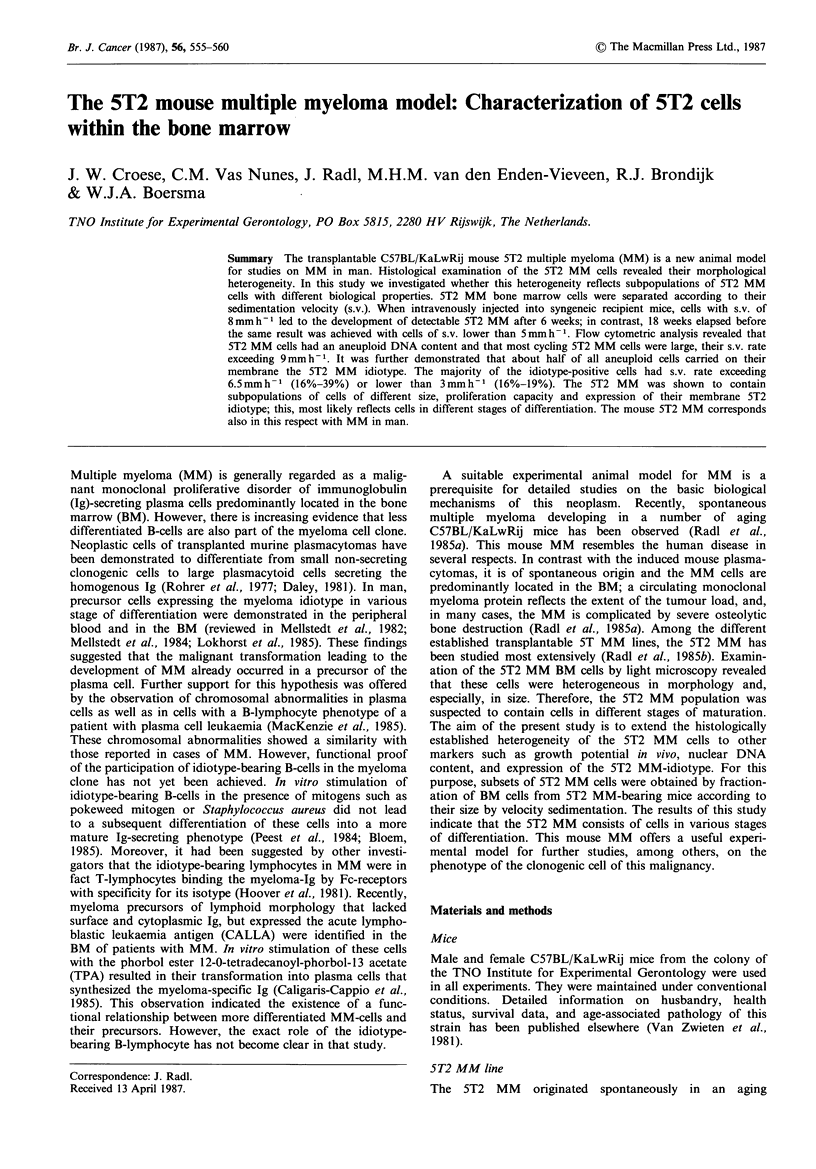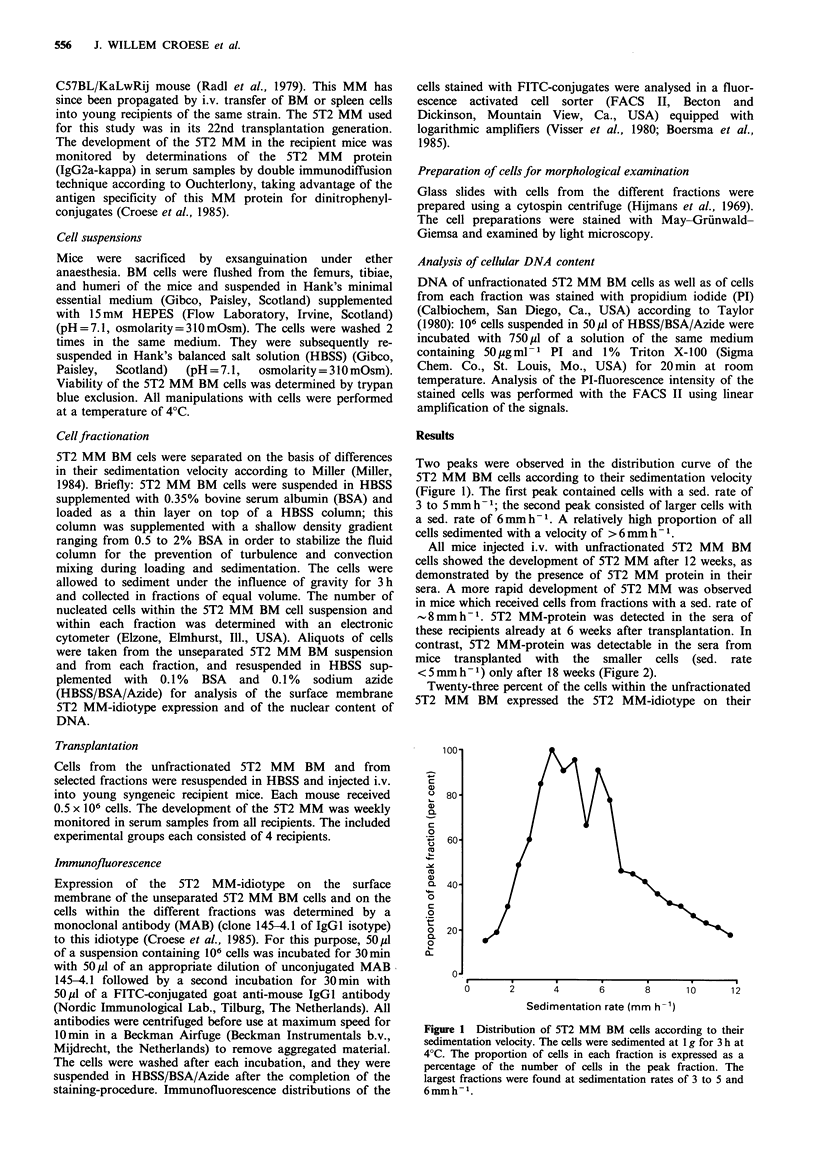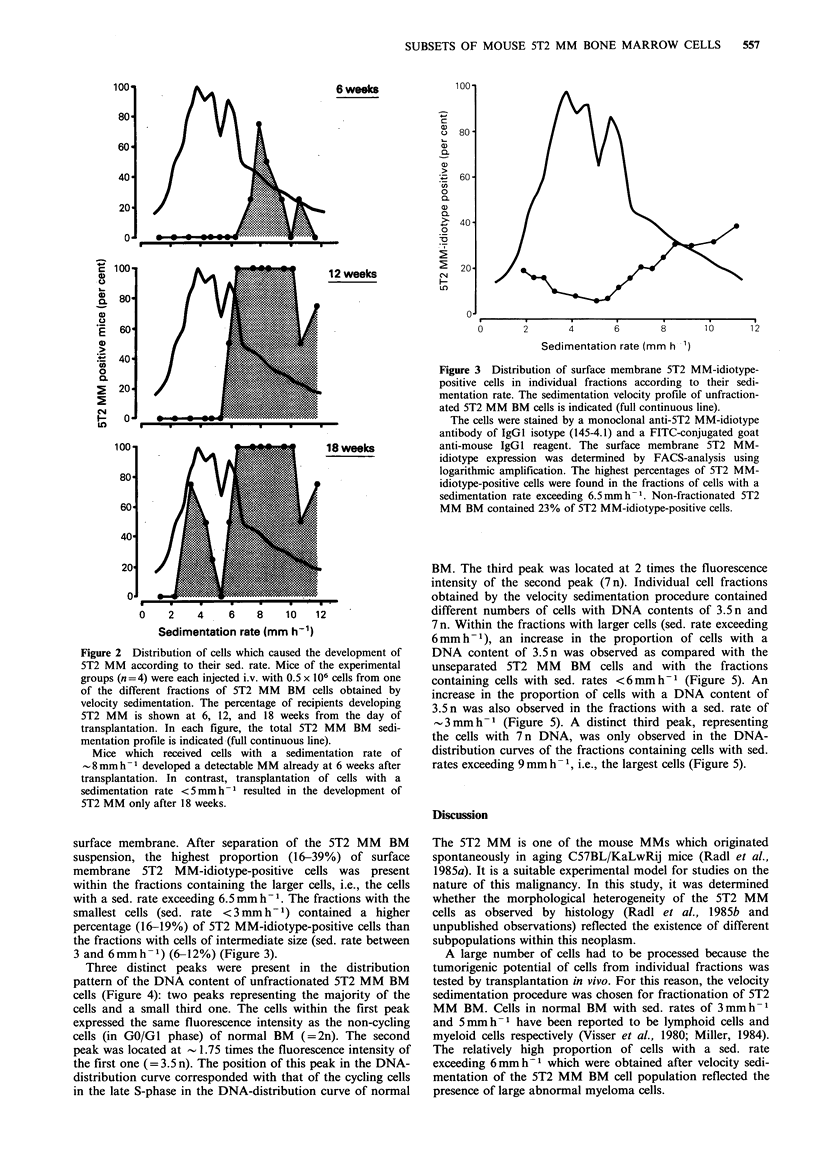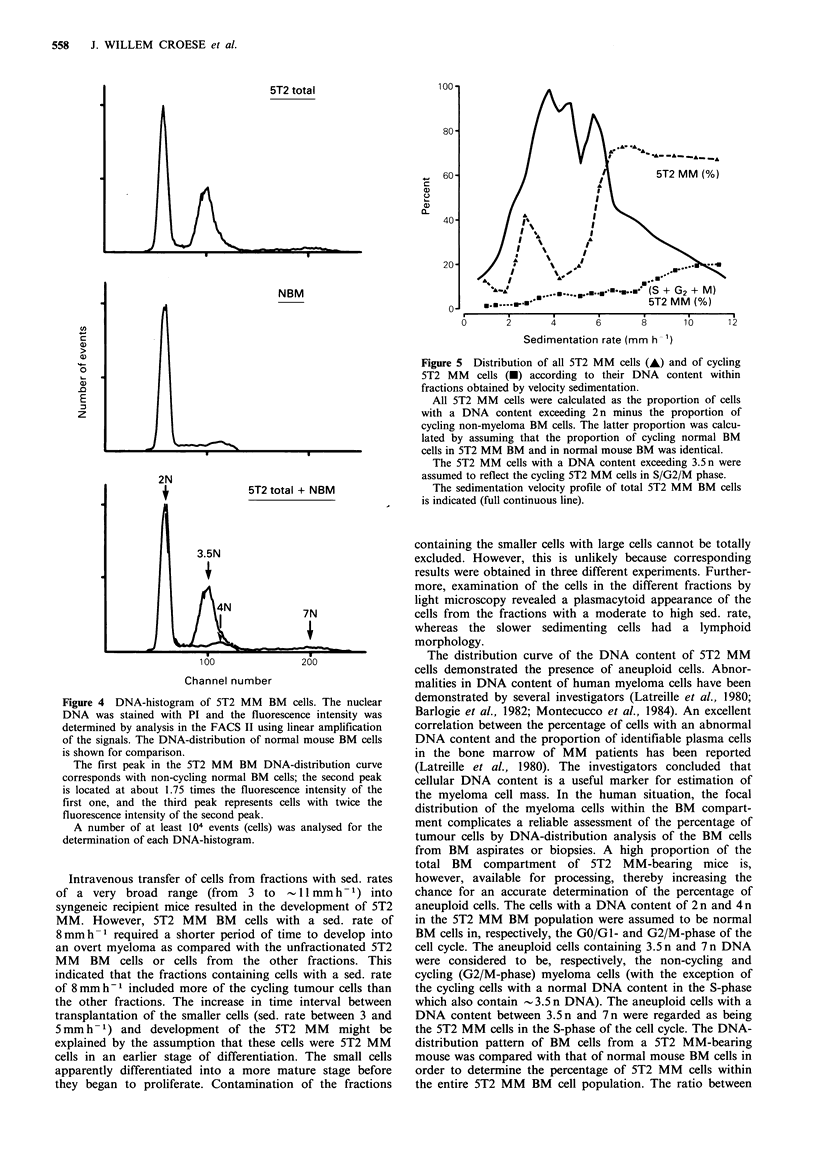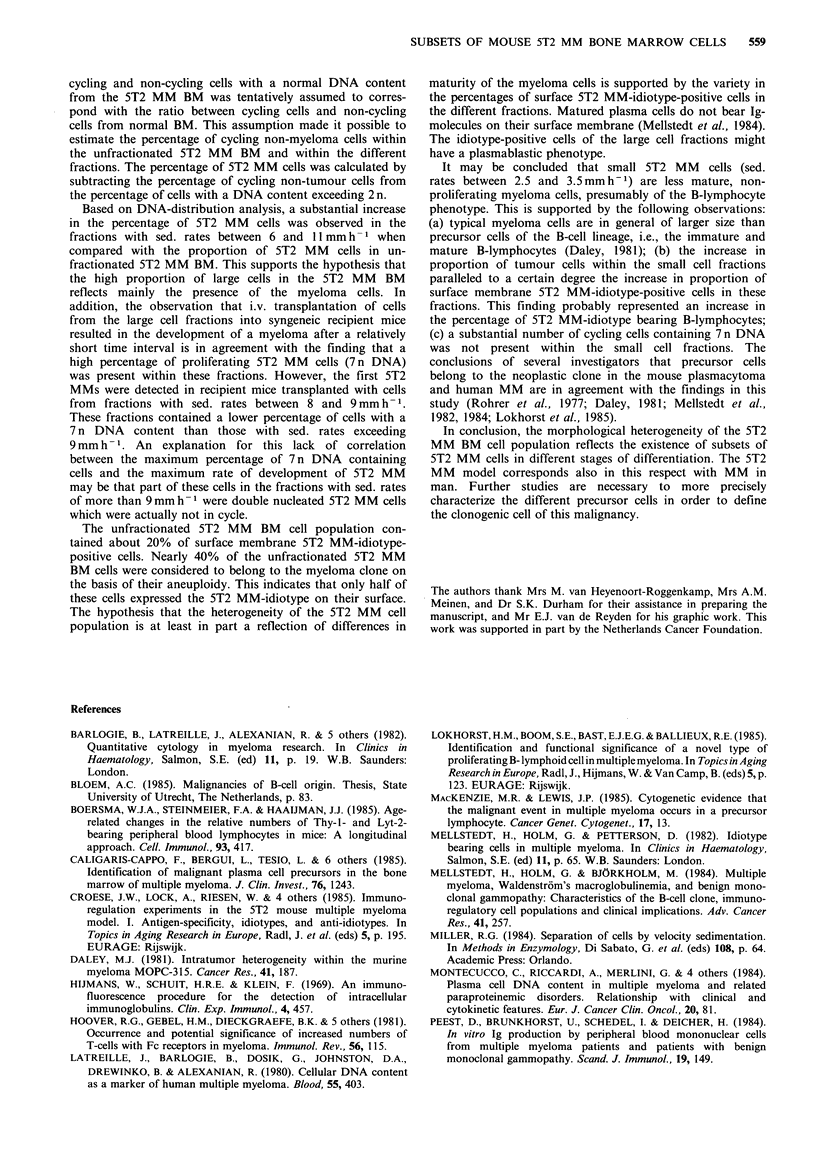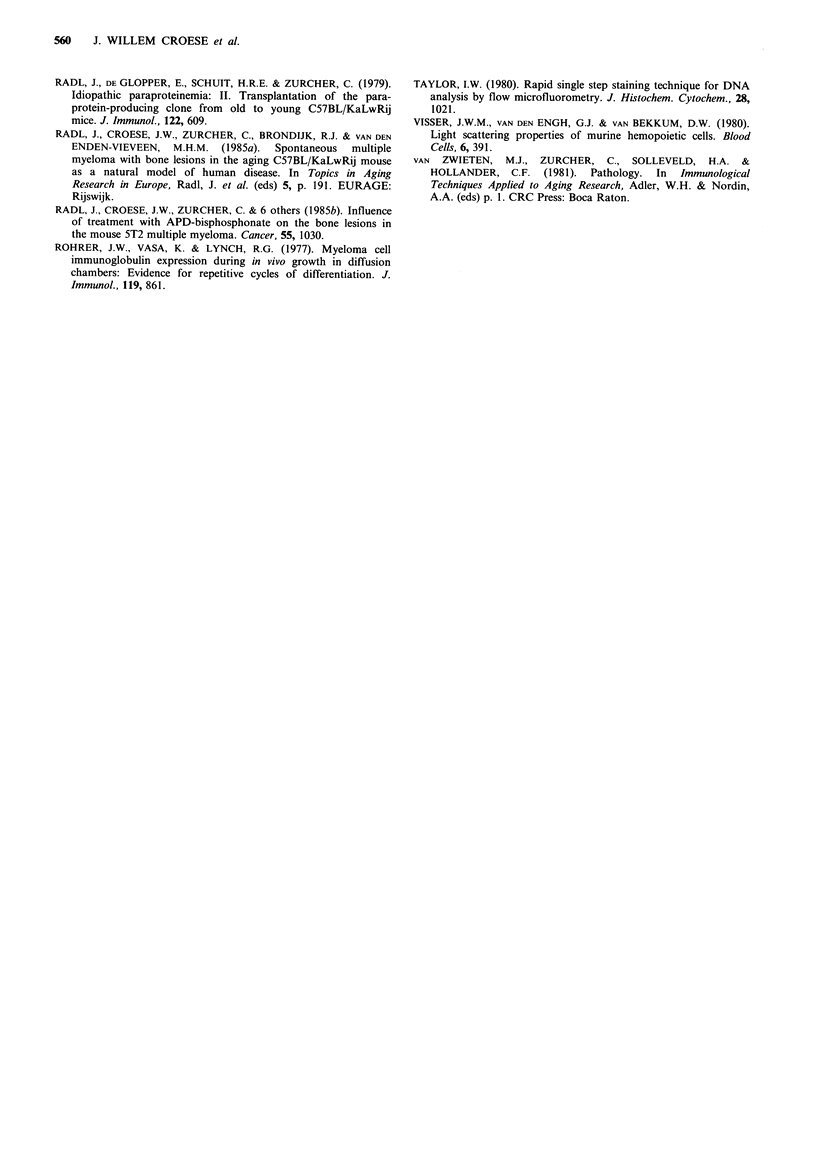# The 5T2 mouse multiple myeloma model: characterization of 5T2 cells within the bone marrow.

**DOI:** 10.1038/bjc.1987.241

**Published:** 1987-11

**Authors:** J. W. Croese, C. M. Vas Nunes, J. Radl, M. H. van den Enden-Vieveen, R. J. Brondijk, W. J. Boersma

**Affiliations:** TNO Institute for Experimental Gerontology, Rijswijk, The Netherlands.

## Abstract

The transplantable C57BL/KaLwRij mouse 5T2 multiple myeloma (MM) is a new animal model for studies on MM in man. Histological examination of the 5T2 MM cells revealed their morphological heterogeneity. In this study we investigated whether this heterogeneity reflects subpopulations of 5T2 MM cells with different biological properties. 5T2 MM bone marrow cells were separated according to their sedimentation velocity (s.v.). When intravenously injected into syngeneic recipient mice, cells with s.v. of 8 mm h-1 led to the development of detectable 5T2 MM after 6 weeks; in contrast, 18 weeks elapsed before the same result was achieved with cells of s.v. lower than 5 mm h-1. Flow cytometric analysis revealed that 5T2 MM cells had an aneuploid DNA content and that most cycling 5T2 MM cells were large, their s.v. rate exceeding 9 mm h-1. It was further demonstrated that about half of all aneuploid cells carried on their membrane the 5T2 MM idiotype. The majority of the idiotype-positive cells had s.v. rate exceeding 6.5 mm h-1 (16%-39%) or lower than 3 mm h-1 (16%-19%). The 5T2 MM was shown to contain subpopulations of cells of different size, proliferation capacity and expression of their membrane 5T2 idiotype; this, most likely reflects cells in different stages of differentiation. The mouse 5T2 MM corresponds also in this respect with MM in man.


*Br. J. Cancer* (1987), **56**, 555–560 © The Macmillan Press Ltd., 1987

### The 5T2 mouse multiple myeloma model: Characterization of 5T2 cells within the bone marrow

J. W. Croese, C.M. Vas Nunes, J. Radl, M.H.M. van den Enden-Vieveen, R.J. Brondijk & W.J.A. Boersma

*TNO Institute for Experimental Gerontology, PO Box 5815, 2280 HV Rijswijk, The Netherlands.*

**Summary** The transplantable C57BL/KaLwRij mouse 5T2 multiple myeloma (MM) is a new animal model for studies on MM in man. Histological examination of the 5T2 MM cells revealed their morphological heterogeneity. In this study we investigated whether this heterogeneity reflects subpopulations of 5T2 MM cells with different biological properties. 5T2 MM bone marrow cells were separated according to their sedimentation velocity (s.v.). When intravenously injected into syngeneic recipient mice, cells with s.v. of $8\,mm\,h^{-1}$ led to the development of detectable 5T2 MM after 6 weeks; in contrast, 18 weeks elapsed before the same result was achieved with cells of s.v. lower than $5\,mm\,h^{-1}$. Flow cytometric analysis revealed that 5T2 MM cells had an aneuploid DNA content and that most cycling 5T2 MM cells were large, their s.v. rate exceeding $9\,mm\,h^{-1}$. It was further demonstrated that about half of all aneuploid cells carried on their membrane the 5T2 MM idiotype. The majority of the idiotype-positive cells had s.v. rate exceeding $6.5\,mm\,h^{-1}$ (16%–39%) or lower than $3\,mm\,h^{-1}$ (16%–19%). The 5T2 MM was shown to contain subpopulations of cells of different size, proliferation capacity and expression of their membrane 5T2 idiotype; this, most likely reflects cells in different stages of differentiation. The mouse 5T2 MM corresponds also in this respect with MM in man.

Multiple myeloma (MM) is generally regarded as a malignant monoclonal proliferative disorder of immunoglobulin (Ig)-secreting plasma cells predominantly located in the bone marrow (BM). However, there is increasing evidence that less differentiated B-cells are also part of the myeloma cell clone. Neoplastic cells of transplanted murine plasmacytomas have been demonstrated to differentiate from small non-secreting clonogenic cells to large plasmacytoid cells secreting the homogenous Ig (Rohrer *et al.*, 1977; Daley, 1981). In man, precursor cells expressing the myeloma idiotype in various stage of differentiation were demonstrated in the peripheral blood and in the BM (reviewed in Mellstedt *et al.*, 1982; Mellstedt *et al.*, 1984; Lokhorst *et al.*, 1985). These findings suggested that the malignant transformation leading to the development of MM already occurred in a precursor of the plasma cell. Further support for this hypothesis was offered by the observation of chromosomal abnormalities in plasma cells as well as in cells with a B-lymphocyte phenotype of a patient with plasma cell leukaemia (MacKenzie *et al.*, 1985). These chromosomal abnormalities showed a similarity with those reported in cases of MM. However, functional proof of the participation of idiotype-bearing B-cells in the myeloma clone has not yet been achieved. *In vitro* stimulation of idiotype-bearing B-cells in the presence of mitogens such as pokeweed mitogen or *Staphylococcus aureus* did not lead to a subsequent differentiation of these cells into a more mature Ig-secreting phenotype (Peest *et al.*, 1984; Bloem, 1985). Moreover, it had been suggested by other investigators that the idiotype-bearing lymphocytes in MM were in fact T-lymphocytes binding the myeloma-Ig by Fc-receptors with specificity for its isotype (Hoover *et al.*, 1981). Recently, myeloma precursors of lymphoid morphology that lacked surface and cytoplasmic Ig, but expressed the acute lymphoblastic leukaemia antigen (CALLA) were identified in the BM of patients with MM. *In vitro* stimulation of these cells with the phorbol ester 12-0-tetradecanoyl-phorbol-13 acetate (TPA) resulted in their transformation into plasma cells that synthesized the myeloma-specific Ig (Caligaris-Cappio *et al.*, 1985). This observation indicated the existence of a functional relationship between more differentiated MM-cells and their precursors. However, the exact role of the idiotype-bearing B-lymphocyte has not become clear in that study.

Correspondence: J. Radl.
Received 13 April 1987.

A suitable experimental animal model for MM is a prerequisite for detailed studies on the basic biological mechanisms of this neoplasm. Recently, spontaneous multiple myeloma developing in a number of aging C57BL/KaLwRij mice has been observed (Radl *et al.*, 1985*a*). This mouse MM resembles the human disease in several respects. In contrast with the induced mouse plasmacytomas, it is of spontaneous origin and the MM cells are predominantly located in the BM; a circulating monoclonal myeloma protein reflects the extent of the tumour load, and, in many cases, the MM is complicated by severe osteolytic bone destruction (Radl *et al.*, 1985*a*). Among the different established transplantable 5T MM lines, the 5T2 MM has been studied most extensively (Radl *et al.*, 1985*b*). Examination of the 5T2 MM BM cells by light microscopy revealed that these cells were heterogeneous in morphology and, especially, in size. Therefore, the 5T2 MM population was suspected to contain cells in different stages of maturation. The aim of the present study is to extend the histologically established heterogeneity of the 5T2 MM cells to other markers such as growth potential *in vivo*, nuclear DNA content, and expression of the 5T2 MM-idiotype. For this purpose, subsets of 5T2 MM cells were obtained by fractionation of BM cells from 5T2 MM-bearing mice according to their size by velocity sedimentation. The results of this study indicate that the 5T2 MM consists of cells in various stages of differentiation. This mouse MM offers a useful experimental model for further studies, among others, on the phenotype of the clonogenic cell of this malignancy.

### Materials and methods

### *Mice*

Male and female C57BL/KaLwRij mice from the colony of the TNO Institute for Experimental Gerontology were used in all experiments. They were maintained under conventional conditions. Detailed information on husbandry, health status, survival data, and age-associated pathology of this strain has been published elsewhere (Van Zwieten *et al.*, 1981).

### *5T2 MM line*

The 5T2 MM originated spontaneously in an aging

C57BL/KaLwRij mouse (Radl *et al.*, 1979). This MM has since been propagated by i.v. transfer of BM or spleen cells into young recipients of the same strain. The 5T2 MM used for this study was in its 22nd transplantation generation. The development of the 5T2 MM in the recipient mice was monitored by determinations of the 5T2 MM protein (IgG2a-kappa) in serum samples by double immunodiffusion technique according to Ouchterlony, taking advantage of the antigen specificity of this MM protein for dinitrophenyl-conjugates (Croese *et al.*, 1985).

*Cell suspensions*

Mice were sacrificed by exsanguination under ether anaesthesia. BM cells were flushed from the femurs, tibiae, and humeri of the mice and suspended in Hank's minimal essential medium (Gibco, Paisley, Scotland) supplemented with 15 mM HEPES (Flow Laboratory, Irvine, Scotland) (pH = 7.1, osmolarity = 310 mOsm). The cells were washed 2 times in the same medium. They were subsequently re-suspended in Hank's balanced salt solution (HBSS) (Gibco, Paisley, Scotland) (pH = 7.1, osmolarity = 310 mOsm). Viability of the 5T2 MM BM cells was determined by trypan blue exclusion. All manipulations with cells were performed at a temperature of 4°C.

*Cell fractionation*

5T2 MM BM cels were separated on the basis of differences in their sedimentation velocity according to Miller (Miller, 1984). Briefly: 5T2 MM BM cells were suspended in HBSS supplemented with 0.35% bovine serum albumin (BSA) and loaded as a thin layer on top of a HBSS column; this column was supplemented with a shallow density gradient ranging from 0.5 to 2% BSA in order to stabilize the fluid column for the prevention of turbulence and convection mixing during loading and sedimentation. The cells were allowed to sediment under the influence of gravity for 3 h and collected in fractions of equal volume. The number of nucleated cells within the 5T2 MM BM cell suspension and within each fraction was determined with an electronic cytometer (Elzone, Elmhurst, Ill., USA). Aliquots of cells were taken from the unseparated 5T2 MM BM suspension and from each fraction, and resuspended in HBSS supplemented with 0.1% BSA and 0.1% sodium azide (HBSS/BSA/Azide) for analysis of the surface membrane 5T2 MM-idiotype expression and of the nuclear content of DNA.

*Transplantation*

Cells from the unfractionated 5T2 MM BM and from selected fractions were resuspended in HBSS and injected i.v. into young syngeneic recipient mice. Each mouse received $0.5 \times 10^6$ cells. The development of the 5T2 MM was weekly monitored in serum samples from all recipients. The included experimental groups each consisted of 4 recipients.

*Immunofluorescence*

Expression of the 5T2 MM-idiotype on the surface membrane of the unseparated 5T2 MM BM cells and on the cells within the different fractions was determined by a monoclonal antibody (MAB) (clone 145–4.1 of IgG1 isotype) to this idiotype (Croese *et al.*, 1985). For this purpose, 50 $\mu$l of a suspension containing $10^6$ cells was incubated for 30 min with 50 $\mu$l of an appropriate dilution of unconjugated MAB 145–4.1 followed by a second incubation for 30 min with 50 $\mu$l of a FITC-conjugated goat anti-mouse IgG1 antibody (Nordic Immunological Lab., Tilburg, The Netherlands). All antibodies were centrifuged before use at maximum speed for 10 min in a Beckman Airfuge (Beckman Instrumentals b.v., Mijdrecht, the Netherlands) to remove aggregated material. The cells were washed after each incubation, and they were suspended in HBSS/BSA/Azide after the completion of the staining-procedure. Immunofluorescence distributions of the cells stained with FITC-conjugates were analysed in a fluorescence activated cell sorter (FACS II, Becton and Dickinson, Mountain View, Ca., USA) equipped with logarithmic amplifiers (Visser *et al.*, 1980; Boersma *et al.*, 1985).

*Preparation of cells for morphological examination*

Glass slides with cells from the different fractions were prepared using a cytospin centrifuge (Hijmans *et al.*, 1969). The cell preparations were stained with May–Grünwald–Giemsa and examined by light microscopy.

*Analysis of cellular DNA content*

DNA of unfractionated 5T2 MM BM cells as well as of cells from each fraction was stained with propidium iodide (PI) (Calbiochem, San Diego, Ca., USA) according to Taylor (1980): $10^6$ cells suspended in 50 $\mu$l of HBSS/BSA/Azide were incubated with 750 $\mu$l of a solution of the same medium containing 50 $\mu$g ml$^{-1}$ PI and 1% Triton X-100 (Sigma Chem. Co., St. Louis, Mo., USA) for 20 min at room temperature. Analysis of the PI-fluorescence intensity of the stained cells was performed with the FACS II using linear amplification of the signals.

**Results**

Two peaks were observed in the distribution curve of the 5T2 MM BM cells according to their sedimentation velocity (Figure 1). The first peak contained cells with a sed. rate of 3 to 5 mm h$^{-1}$; the second peak consisted of larger cells with a sed. rate of 6 mm h$^{-1}$. A relatively high proportion of all cells sedimented with a velocity of $>$6 mm h$^{-1}$.

All mice injected i.v. with unfractionated 5T2 MM BM cells showed the development of 5T2 MM after 12 weeks, as demonstrated by the presence of 5T2 MM protein in their sera. A more rapid development of 5T2 MM was observed in mice which received cells from fractions with a sed. rate of $\sim$8 mm h$^{-1}$. 5T2 MM-protein was detected in the sera of these recipients already at 6 weeks after transplantation. In contrast, 5T2 MM-protein was detectable in the sera from mice transplanted with the smaller cells (sed. rate $<$5 mm h$^{-1}$) only after 18 weeks (Figure 2).

Twenty-three percent of the cells within the unfractionated 5T2 MM BM expressed the 5T2 MM-idiotype on their

**Figure 1** Distribution of 5T2 MM BM cells according to their sedimentation velocity. The cells were sedimented at 1 *g* for 3 h at 4°C. The proportion of cells in each fraction is expressed as a percentage of the number of cells in the peak fraction. The largest fractions were found at sedimentation rates of 3 to 5 and 6 mm h$^{-1}$.

**Figure 2** Distribution of cells which caused the development of 5T2 MM according to their sed. rate. Mice of the experimental groups ($n=4$) were each injected i.v. with $0.5 \times 10^6$ cells from one of the different fractions of 5T2 MM BM cells obtained by velocity sedimentation. The percentage of recipients developing 5T2 MM is shown at 6, 12, and 18 weeks from the day of transplantation. In each figure, the total 5T2 MM BM sedimentation profile is indicated (full continuous line).

Mice which received cells with a sedimentation rate of $\sim 8\,\text{mm}\,\text{h}^{-1}$ developed a detectable MM already at 6 weeks after transplantation. In contrast, transplantation of cells with a sedimentation rate $< 5\,\text{mm}\,\text{h}^{-1}$ resulted in the development of 5T2 MM only after 18 weeks.

**Figure 3** Distribution of surface membrane 5T2 MM-idiotype-positive cells in individual fractions according to their sedimentation rate. The sedimentation velocity profile of unfractionated 5T2 MM BM cells is indicated (full continuous line).

The cells were stained by a monoclonal anti-5T2 MM-idiotype antibody of IgG1 isotype (145-4.1) and a FITC-conjugated goat anti-mouse IgG1 reagent. The surface membrane 5T2 MM-idiotype expression was determined by FACS-analysis using logarithmic amplification. The highest percentages of 5T2 MM-idiotype-positive cells were found in the fractions of cells with a sedimentation rate exceeding $6.5\,\text{mm}\,\text{h}^{-1}$. Non-fractionated 5T2 MM BM contained 23% of 5T2 MM-idiotype-positive cells.

surface membrane. After separation of the 5T2 MM BM suspension, the highest proportion (16–39%) of surface membrane 5T2 MM-idiotype-positive cells was present within the fractions containing the larger cells, i.e., the cells with a sed. rate exceeding $6.5\,\text{mm}\,\text{h}^{-1}$. The fractions with the smallest cells (sed. rate $< 3\,\text{mm}\,\text{h}^{-1}$) contained a higher percentage (16–19%) of 5T2 MM-idiotype-positive cells than the fractions with cells of intermediate size (sed. rate between 3 and $6\,\text{mm}\,\text{h}^{-1}$) (6–12%) (Figure 3).

Three distinct peaks were present in the distribution pattern of the DNA content of unfractionated 5T2 MM BM cells (Figure 4): two peaks representing the majority of the cells and a small third one. The cells within the first peak expressed the same fluorescence intensity as the non-cycling cells (in G0/G1 phase) of normal BM (=2n). The second peak was located at ~1.75 times the fluorescence intensity of the first one (=3.5 n). The position of this peak in the DNA-distribution curve corresponded with that of the cycling cells in the late S-phase in the DNA-distribution curve of normal BM. The third peak was located at 2 times the fluorescence intensity of the second peak (7 n). Individual cell fractions obtained by the velocity sedimentation procedure contained different numbers of cells with DNA contents of 3.5 n and 7 n. Within the fractions with larger cells (sed. rate exceeding $6\,\text{mm}\,\text{h}^{-1}$), an increase in the proportion of cells with a DNA content of 3.5 n was observed as compared with the unseparated 5T2 MM BM cells and with the fractions containing cells with sed. rates $< 6\,\text{mm}\,\text{h}^{-1}$ (Figure 5). An increase in the proportion of cells with a DNA content of 3.5 n was also observed in the fractions with a sed. rate of $\sim 3\,\text{mm}\,\text{h}^{-1}$ (Figure 5). A distinct third peak, representing the cells with 7 n DNA, was only observed in the DNA-distribution curves of the fractions containing cells with sed. rates exceeding $9\,\text{mm}\,\text{h}^{-1}$, i.e., the largest cells (Figure 5).

### Discussion

The 5T2 MM is one of the mouse MMs which originated spontaneously in aging C57BL/KaLwRij mice (Radl *et al.*, 1985*a*). It is a suitable experimental model for studies on the nature of this malignancy. In this study, it was determined whether the morphological heterogeneity of the 5T2 MM cells as observed by histology (Radl *et al.*, 1985*b* and unpublished observations) reflected the existence of different subpopulations within this neoplasm.

A large number of cells had to be processed because the tumorigenic potential of cells from individual fractions was tested by transplantation *in vivo*. For this reason, the velocity sedimentation procedure was chosen for fractionation of 5T2 MM BM. Cells in normal BM with sed. rates of $3\,\text{mm}\,\text{h}^{-1}$ and $5\,\text{mm}\,\text{h}^{-1}$ have been reported to be lymphoid cells and myeloid cells respectively (Visser *et al.*, 1980; Miller, 1984). The relatively high proportion of cells with a sed. rate exceeding $6\,\text{mm}\,\text{h}^{-1}$ which were obtained after velocity sedimentation of the 5T2 MM BM cell population reflected the presence of large abnormal myeloma cells.

**Figure 4** DNA-histogram of 5T2 MM BM cells. The nuclear DNA was stained with PI and the fluorescence intensity was determined by analysis in the FACS II using linear amplification of the signals. The DNA-distribution of normal mouse BM cells is shown for comparison.

The first peak in the 5T2 MM BM DNA-distribution curve corresponds with non-cycling normal BM cells; the second peak is located at about 1.75 times the fluorescence intensity of the first one, and the third peak represents cells with twice the fluorescence intensity of the second peak.

A number of at least $10^4$ events (cells) was analysed for the determination of each DNA-histogram.

**Figure 5** Distribution of all 5T2 MM cells (▲) and of cycling 5T2 MM cells (■) according to their DNA content within fractions obtained by velocity sedimentation.

All 5T2 MM cells were calculated as the proportion of cells with a DNA content exceeding 2n minus the proportion of cycling non-myeloma BM cells. The latter proportion was calculated by assuming that the proportion of cycling normal BM cells in 5T2 MM BM and in normal mouse BM was identical.

The 5T2 MM cells with a DNA content exceeding 3.5n were assumed to reflect the cycling 5T2 MM cells in S/G2/M phase.

The sedimentation velocity profile of total 5T2 MM BM cells is indicated (full continuous line).

Intravenous transfer of cells from fractions with sed. rates of a very broad range (from 3 to $\sim 11\,\text{mm}\,\text{h}^{-1}$) into syngeneic recipient mice resulted in the development of 5T2 MM. However, 5T2 MM BM cells with a sed. rate of $8\,\text{mm}\,\text{h}^{-1}$ required a shorter period of time to develop into an overt myeloma as compared with the unfractionated 5T2 MM BM cells or cells from the other fractions. This indicated that the fractions containing cells with a sed. rate of $8\,\text{mm}\,\text{h}^{-1}$ included more of the cycling tumour cells than the other fractions. The increase in time interval between transplantation of the smaller cells (sed. rate between 3 and $5\,\text{mm}\,\text{h}^{-1}$) and development of the 5T2 MM might be explained by the assumption that these cells were 5T2 MM cells in an earlier stage of differentiation. The small cells apparently differentiated into a more mature stage before they began to proliferate. Contamination of the fractions containing the smaller cells with large cells cannot be totally excluded. However, this is unlikely because corresponding results were obtained in three different experiments. Furthermore, examination of the cells in the different fractions by light microscopy revealed a plasmacytoid appearance of the cells from the fractions with a moderate to high sed. rate, whereas the slower sedimenting cells had a lymphoid morphology.

The distribution curve of the DNA content of 5T2 MM cells demonstrated the presence of aneuploid cells. Abnormalities in DNA content of human myeloma cells have been demonstrated by several investigators (Latreille *et al.*, 1980; Barlogie *et al.*, 1982; Montecucco *et al.*, 1984). An excellent correlation between the percentage of cells with an abnormal DNA content and the proportion of identifiable plasma cells in the bone marrow of MM patients has been reported (Latreille *et al.*, 1980). The investigators concluded that cellular DNA content is a useful marker for estimation of the myeloma cell mass. In the human situation, the focal distribution of the myeloma cells within the BM compartment complicates a reliable assessment of the percentage of tumour cells by DNA-distribution analysis of the BM cells from BM aspirates or biopsies. A high proportion of the total BM compartment of 5T2 MM-bearing mice is, however, available for processing, thereby increasing the chance for an accurate determination of the percentage of aneuploid cells. The cells with a DNA content of 2n and 4n in the 5T2 MM BM population were assumed to be normal BM cells in, respectively, the G0/G1- and G2/M-phase of the cell cycle. The aneuploid cells containing 3.5n and 7n DNA were considered to be, respectively, the non-cycling and cycling (G2/M-phase) myeloma cells (with the exception of the cycling cells with a normal DNA content in the S-phase which also contain ~3.5n DNA). The aneuploid cells with a DNA content between 3.5n and 7n were regarded as being the 5T2 MM cells in the S-phase of the cell cycle. The DNA-distribution pattern of BM cells from a 5T2 MM-bearing mouse was compared with that of normal mouse BM cells in order to determine the percentage of 5T2 MM cells within the entire 5T2 MM BM cell population. The ratio between cycling and non-cycling cells with a normal DNA content from the 5T2 MM BM was tentatively assumed to correspond with the ratio between cycling cells and non-cycling cells from normal BM. This assumption made it possible to estimate the percentage of cycling non-myeloma cells within the unfractionated 5T2 MM BM and within the different fractions. The percentage of 5T2 MM cells was calculated by subtracting the percentage of cycling non-tumour cells from the percentage of cells with a DNA content exceeding 2 n.

Based on DNA-distribution analysis, a substantial increase in the percentage of 5T2 MM cells was observed in the fractions with sed. rates between 6 and 11 mm $h^{-1}$ when compared with the proportion of 5T2 MM cells in unfractionated 5T2 MM BM. This supports the hypothesis that the high proportion of large cells in the 5T2 MM BM reflects mainly the presence of the myeloma cells. In addition, the observation that i.v. transplantation of cells from the large cell fractions into syngeneic recipient mice resulted in the development of a myeloma after a relatively short time interval is in agreement with the finding that a high percentage of proliferating 5T2 MM cells (7 n DNA) was present within these fractions. However, the first 5T2 MMs were detected in recipient mice transplanted with cells from fractions with sed. rates between 8 and 9 mm $h^{-1}$. These fractions contained a lower percentage of cells with a 7 n DNA content than those with sed. rates exceeding 9 mm $h^{-1}$. An explanation for this lack of correlation between the maximum percentage of 7 n DNA containing cells and the maximum rate of development of 5T2 MM may be that part of these cells in the fractions with sed. rates of more than 9 mm $h^{-1}$ were double nucleated 5T2 MM cells which were actually not in cycle.

The unfractionated 5T2 MM BM cell population contained about 20% of surface membrane 5T2 MM-idiotype-positive cells. Nearly 40% of the unfractionated 5T2 MM BM cells were considered to belong to the myeloma clone on the basis of their aneuploidy. This indicates that only half of these cells expressed the 5T2 MM-idiotype on their surface. The hypothesis that the heterogeneity of the 5T2 MM cell population is at least in part a reflection of differences in maturity of the myeloma cells is supported by the variety in the percentages of surface 5T2 MM-idiotype-positive cells in the different fractions. Matured plasma cells do not bear Ig-molecules on their surface membrane (Mellstedt *et al.*, 1984). The idiotype-positive cells of the large cell fractions might have a plasmablastic phenotype.

It may be concluded that small 5T2 MM cells (sed. rates between 2.5 and 3.5 mm $h^{-1}$) are less mature, non-proliferating myeloma cells, presumably of the B-lymphocyte phenotype. This is supported by the following observations: (a) typical myeloma cells are in general of larger size than precursor cells of the B-cell lineage, i.e., the immature and mature B-lymphocytes (Daley, 1981); (b) the increase in proportion of tumour cells within the small cell fractions paralleled to a certain degree the increase in proportion of surface membrane 5T2 MM-idiotype-positive cells in these fractions. This finding probably represented an increase in the percentage of 5T2 MM-idiotype bearing B-lymphocytes; (c) a substantial number of cycling cells containing 7 n DNA was not present within the small cell fractions. The conclusions of several investigators that precursor cells belong to the neoplastic clone in the mouse plasmacytoma and human MM are in agreement with the findings in this study (Rohrer *et al.*, 1977; Daley, 1981; Mellstedt *et al.*, 1982, 1984; Lokhorst *et al.*, 1985).

In conclusion, the morphological heterogeneity of the 5T2 MM BM cell population reflects the existence of subsets of 5T2 MM cells in different stages of differentiation. The 5T2 MM model corresponds also in this respect with MM in man. Further studies are necessary to more precisely characterize the different precursor cells in order to define the clonogenic cell of this malignancy.

The authors thank Mrs M. van Heyenoort-Roggenkamp, Mrs A.M. Meinen, and Dr S.K. Durham for their assistance in preparing the manuscript, and Mr E.J. van de Reyden for his graphic work. This work was supported in part by the Netherlands Cancer Foundation.

### References

BARLOGIE, B., LATREILLE, J., ALEXANIAN, R. & 5 others (1982). Quantitative cytology in myeloma research. In *Clinics in Haematology*, Salmon, S.E. (ed) **11**, p. 19. W.B. Saunders: London.

BLOEM, A.C. (1985). Malignancies of B-cell origin. Thesis, State University of Utrecht, The Netherlands, p. 83.

BOERSMA, W.J.A., STEINMEIER, F.A. & HAAIJMAN, J.J. (1985). Age-related changes in the relative numbers of Thy-1- and Lyt-2-bearing peripheral blood lymphocytes in mice: A longitudinal approach. *Cell. Immunol.*, **93**, 417.

CALIGARIS-CAPPO, F., BERGUI, L., TESIO, L. & 6 others (1985). Identification of malignant plasma cell precursors in the bone marrow of multiple myeloma. *J. Clin. Invest.*, **76**, 1243.

CROESE, J.W., LOCK, A., RIESEN, W. & 4 others (1985). Immunoregulation experiments in the 5T2 mouse multiple myeloma model. I. Antigen-specificity, idiotypes, and anti-idiotypes. In *Topics in Aging Research in Europe*, Radl, J. *et al.* (eds) **5**, p. 195. EURAGE: Rijswijk.

DALEY, M.J. (1981). Intratumor heterogeneity within the murine myeloma MOPC-315. *Cancer Res.*, **41**, 187.

HIJMANS, W., SCHUIT, H.R.E. & KLEIN, F. (1969). An immunofluorescence procedure for the detection of intracellular immunoglobulins. *Clin. Exp. Immunol.*, **4**, 457.

HOOVER, R.G., GEBEL, H.M., DIECKGRAEFE, B.K. & 5 others (1981). Occurrence and potential significance of increased numbers of T-cells with Fc receptors in myeloma. *Immunol. Rev.*, **56**, 115.

LATREILLE, J., BARLOGIE, B., DOSIK, G., JOHNSTON, D.A., DREWINKO, B. & ALEXANIAN, R. (1980). Cellular DNA content as a marker of human multiple myeloma. *Blood*, **55**, 403.

LOKHORST, H.M., BOOM, S.E., BAST, E.J.E.G. & BALLIEUX, R.E. (1985). Identification and functional significance of a novel type of proliferating B-lymphoid cell in multiple myeloma. In *Topics in Aging Research in Europe*, Radl, J., Hijmans, W. & Van Camp, B. (eds) **5**, p. 123. EURAGE: Rijswijk.

MACKENZIE, M.R. & LEWIS, J.P. (1985). Cytogenetic evidence that the malignant event in multiple myeloma occurs in a precursor lymphocyte. *Cancer Genet. Cytogenet.*, **17**, 13.

MELLSTEDT, H., HOLM, G. & PETTERSON, D. (1982). Idiotype bearing cells in multiple myeloma. In *Clinics in Haematology*, Salmon, S.E. (ed) **11**, p. 65. W.B. Saunders: London.

MELLSTEDT, H., HOLM, G. & BJÖRKHOLM, M. (1984). Multiple myeloma, Waldenström's macroglobulinemia, and benign monoclonal gammopathy: Characteristics of the B-cell clone, immunoregulatory cell populations and clinical implications. *Adv. Cancer Res.*, **41**, 257.

MILLER, R.G. (1984). Separation of cells by velocity sedimentation. In *Methods in Enzymology*, Di Sabato, G. *et al.* (eds) **108**, p. 64. Academic Press: Orlando.

MONTECUCCO, C., RICCARDI, A., MERLINI, G. & 4 others (1984). Plasma cell DNA content in multiple myeloma and related paraproteinemic disorders. Relationship with clinical and cytokinetic features. *Eur. J. Cancer Clin. Oncol.*, **20**, 81.

PEEST, D., BRUNKHORST, U., SCHEDEL, I. & DEICHER, H. (1984). *In vitro* Ig production by peripheral blood mononuclear cells from multiple myeloma patients and patients with benign monoclonal gammopathy. *Scand. J. Immunol.*, **19**, 149.

RADL, J., DE GLOPPER, E., SCHUIT, H.R.E. & ZURCHER, C. (1979). Idiopathic paraproteinemia: II. Transplantation of the paraprotein-producing clone from old to young C57BL/KaLwRij mice. *J. Immunol.*, **122**, 609.

RADL, J., CROESE, J.W., ZURCHER, C., BRONDIJK, R.J. & VAN DEN ENDEN-VIEVEEN, M.H.M. (1985*a*). Spontaneous multiple myeloma with bone lesions in the aging C57BL/KaLwRij mouse as a natural model of human disease. In *Topics in Aging Research in Europe*, Radl, J. *et al.* (eds) **5**, p. 191. EURAGE: Rijswijk.

RADL, J., CROESE, J.W., ZURCHER, C. & 6 others (1985*b*). Influence of treatment with APD-bisphosphonate on the bone lesions in the mouse 5T2 multiple myeloma. *Cancer*, **55**, 1030.

ROHRER, J.W., VASA, K. & LYNCH, R.G. (1977). Myeloma cell immunoglobulin expression during *in vivo* growth in diffusion chambers: Evidence for repetitive cycles of differentiation. *J. Immunol.*, **119**, 861.

TAYLOR, I.W. (1980). Rapid single step staining technique for DNA analysis by flow microfluorometry. *J. Histochem. Cytochem.*, **28**, 1021.

VISSER, J.W.M., VAN DEN ENGH, G.J. & VAN BEKKUM, D.W. (1980). Light scattering properties of murine hemopoietic cells. *Blood Cells*, **6**, 391.

VAN ZWIETEN, M.J., ZURCHER, C., SOLLEVELD, H.A. & HOLLANDER, C.F. (1981). Pathology. In *Immunological Techniques Applied to Aging Research*, Adler, W.H. & Nordin, A.A. (eds) p. 1. CRC Press: Boca Raton.